# Will COVID-19 become mild, like a cold?

**DOI:** 10.1017/S0950268824001110

**Published:** 2024-10-07

**Authors:** Patrick D. Shaw Stewart

**Affiliations:** Independent scientist

**Keywords:** Pathogenicity of respiratory viruses, pathogenicity of COVID-19, viral evolution, virulence, thermal sensitivity, incubation periods

## Abstract

Several recent studies conclude that an increase in the pathogenicity of SARS-CoV-2 cannot be ruled out. However, it should be noted that SARS-CoV-2 is a ‘direct’ respiratory virus – meaning it is usually spread by the respiratory route but does not routinely pass through the lymphatics like measles and smallpox. Providing its tropism does not change, it will be unique if its pathogenicity does not decrease until it becomes similar to common cold viruses. Ewald noted in the 1980s that respiratory viruses may evolve mildness because their spread benefits from the mobility of their hosts. This review examines factors that usually lower respiratory viruses’ severity, including heat sensitivity (which limits replication in the warmer lungs) and changes to the virus’s surface proteins. Other factors may, however, increase pathogenicity, such as replication in the lymphatic system and spreading via solid surfaces or faecal matter. Furthermore, human activities and political events could increase the harmfulness of SARS-CoV-2, including the following: large-scale testing, especially when the results are delayed; transmission in settings where people are close together and not free to move around; poor hygiene facilities; and social, political, or cultural influences that encourage sick individuals to remain active, including crises such as wars. If we can avoid these eventualities, SARS-CoV-2 is likely to evolve to be milder, although the timescale is uncertain. Observations of influenza-like pandemics suggest it may take around two decades for COVID-19 to become as mild as seasonal colds.

## Introduction: SARS-CoV-2 is a respiratory virus with ‘direct’ transmission

Several recent studies investigated the evolutionary development of severe acute respiratory syndrome coronavirus 2 (SARS-CoV-2), suggested that we should not rule out the possibility that it will become more pathogenic. For example, Markov *et al.* emphasized that SARS-CoV-2 infection frequently led to severe illness or death during the third-week post-infection. Since the contagious period generally spanned from day 2 to day 15, most transmissions (90%) occurred before the average time of death. The authors pointed out that the death of the host occurring after transmission would not reduce the fitness of a viral lineage and concluded that safe infections, mild COVID-19, and a low population mortality and morbidity burden should not be assumed [1]. A separate study by Rochman *et al.* constructed a maximally-general compartment model to chart the global phase space of human respiratory viruses, predicting that SARS-CoV-2 will likely continue to present a substantial disease burden [2]. Roemer *et al.* highlight the potential for a future virus variant to emerge that is more dangerous than the currently circulating Omicron variant. They voiced concern that new variants might emerge through recombination with Delta strains since Delta generally has a higher severity than other variants [3]. While increased pathogenicity cannot be ruled out, comparisons with other respiratory viruses suggest that the likely trajectory of SARS-CoV-2 pathogenicity is downwards. This review investigates the biological and human events that could affect the evolution of pathogenicity in SARS-CoV-2, both positively and negatively.

SARS-CoV-2, like other respiratory viruses, typically spreads via aerosols or droplets emitted when an infected individual talks, coughs, or sneezes. However, these particles cannot remain airborne for extended periods [4]. Therefore, for the virus to spread, infected individuals generally need to be near susceptible individuals. In a study from the 1980s, Ewald pointed out that, beyond preserving the lives of their hosts, pathogens such as respiratory viruses can benefit by ensuring that their hosts remain mobile, helping to spread the virus [5]. Humans and animals often refrain from moving around when sick, and data shows that respiratory viruses are typically much milder than viruses spread by vectors such as biting and bloodsucking insects [5]. Since over 200 serologically distinct RNA and DNA virus species cause common colds, and almost all are mild (except in children and vulnerable individuals), the consistent mildness and lack of complications from colds, in contrast to many other viral illnesses, is remarkable [6]. Walther and Ewald reported the infection fatality rates (IFRs) for nine respiratory viral illnesses, calculated as the mean percentage of deaths per infection [7]. Leaving aside smallpox for the time being, these IFRs ranged from 0% for rhinovirus to 0.010% for influenza, as shown in Table 1. The IFRs for viruses transmitted through contaminated water, sexual contact, animal bites, or insects are frequently much higher. For instance, the human IFRs of untreated yellow fever, hepatitis B, HIV infection, and rabies vary from roughly 1% to almost 100%. For comparison, the IFR of SARS-CoV-2 in England and Australia prior to September 2022 was around 1.5% [8].

**Table 1.** The infection fatality rates and survival of 9 respiratory viruses [7]

Respiratory virus	Genetic material	IFR (M/I) %	Survival in external environment (days)
Smallpox virus	DNA	10	885
Influenza virus	RNA	0.01	13.7
Measles virus	RNA	0.007	4.4
Mumps virus	RNA	0.005	0.9
Parainfluenza virus	RNA	0.004	4.6
Respiratory syncytial virus	RNA	0.003	1.1
Chickenpox virus	DNA	0.003	0.9
Rubella virus	RNA	0.003	0.9
Rhinovirus	RNA	0	2.3

Furthermore, SARS-CoV-2 falls into the category that I refer to here as ‘direct’ respiratory viruses (DRVs). By this I mean its usual replication cycle involves multiplying in the cells that line the upper airways and being transmitted without passing through the lymphatic system or the bloodstream. (This does not imply the virus never reaches the bloodstream, and never attacks other tissues, but that SARS-CoV-2 virions that originate in organs other than the airways are seldom transmitted.) This contrasts with other respiratory viruses, including measles, mumps, rubella, and smallpox, which replicate in (or cause swelling of) the lymphatics and then pass into the bloodstream. The presence of the virus in the bloodstream allows it to reach all the organs of the body, which increases the opportunities for complications, suggesting that such viruses are likely to be more severe than DRVs. This is difficult to confirm with empirical data. Only a few respiratory viruses have been studied in detail, and data on fatality rates is not widely available. All of the respiratory viruses listed in Table 1 apart from rhinovirus are relatively pathogenic and the majority are lymphatic-passaging. Common colds are caused by at least 200 viruses, many of which are not widely studied, because they *are* mild (and/or rare). For example, 88 human adenoviruses in seven species are known, many of which cause respiratory illness [9]. Since most respiratory viruses do *not* pass through the lymphatics, and those that do are among the most pathogenic, we might tentatively conclude that DRVs are usually less pathogenic than measles, smallpox, mumps, etc.

Passage through the lymphatics might benefit the viruses involved in two ways: firstly, the virus can multiply invisibly before the host experiences any symptoms. For example, the measles virus has a relatively long incubation period (8–14 days), and it usually replicates in the respiratory tract, local lymph nodes, spleen, lungs, and thymus gland before any symptoms occur [10]. This may slow up the development of the illness and extend the prime contagious period that lasts until the infected individual becomes too sick to interact with others. Secondly, since the virus suddenly appears all over the lungs and upper airways in great quantities, infected hosts may shed virus particles at a higher rate. The downside for the host (but not usually for the virus) is that such widespread internal infections can be fatal or lead to complications affecting other organs, such as the eyes, ears, and brain in the case of measles, or affecting unborn children in the case of rubella. Since SARS-CoV-2 does not normally pass through the lymphatics or enter the bloodstream before transmission, we might expect its pathogenicity to diminish.

As mentioned above, smallpox, with an IFR of 10%, was uniquely pathogenic among respiratory viruses. Walther and Ewald propose that one contributing factor was the exceptional durability of the virus outside the body; in one instance, it survived in a dried exudate in indirect light for over two years (Table 1) [7]. This durability allowed the virus to adopt a sit-and-wait strategy, making its hosts’ mobility less critical. It also implies that smallpox was often spread by other means, not just through droplets and aerosols, differentiating it from typical respiratory viruses. Influenza, which can survive in the external environment for over 10 days, is also unusually enduring, possibly accounting for its higher-than-normal pathogenicity (Table 1). SARS-CoV-2 is principally transmitted by air, and the airborne virus has a half-life of approximately 3 h [4]. It can also be transmitted via solid surfaces, having a half-life of around 5 h for the ancestral lineage WA1. The early variants of concern Alpha and Beta had slightly longer half-lives of around 5–6 h both in air and on surfaces, but the BA.1 Omicron variant was less stable than WA1. Since these periods are lower than those of the respiratory viruses shown in Table 1, we might anticipate selection for high host mobility and low pathogenicity in the future. However, we cannot rule out the emergence of increased transmission on solid surfaces and selection for greater durability if the mode of transmission changes.

Diarrhoea, sometimes bloody, occurs in approximately 10% to 20% of hospitalized patients with COVID-19 infection, and it is thought that the virus can replicate in the intestinal epithelium [11], opening up the possibility of transmission by the faecal-oral route. We should, therefore, consider the pathogenicity of viruses that are spread by this route. Norovirus typically causes non-bloody diarrhoea, vomiting, and stomach pain, sometimes with fever or headaches. It is spread predominantly through the faecal-oral route, either through contaminated food or surfaces, or directly from person to person [12]. However, it rarely causes complications, and recovery typically takes one to three days. Other viruses spread by the faecal-oral route include several strains of coxsackievirus and enterovirus that cause hand, foot, and mouth disease, mainly in children. Symptoms include skin rashes, showing they reach the bloodstream. However, the disease is normally mild, and most patients recover within 10 days. This mildness may reflect the need for children to be physically close to spread the disease efficiently. Interestingly, the same viruses often cause common colds in adults. Occasionally, hand, foot, and mouth disease from enterovirus 71 causes complications, such as cardiac symptoms, viral meningitis, or encephalitis [13]. Another disease that more often causes complications is polio. Seventy-five per cent of polio infections are asymptomatic, but in a few cases, the virus migrates from the gut to the central nervous system, causing paralysis in 0.1%–1% of cases [14]. Increased transmission of SARS-CoV-2 by the faecal-oral route might be a very unwelcome development.

**Figure 1.**
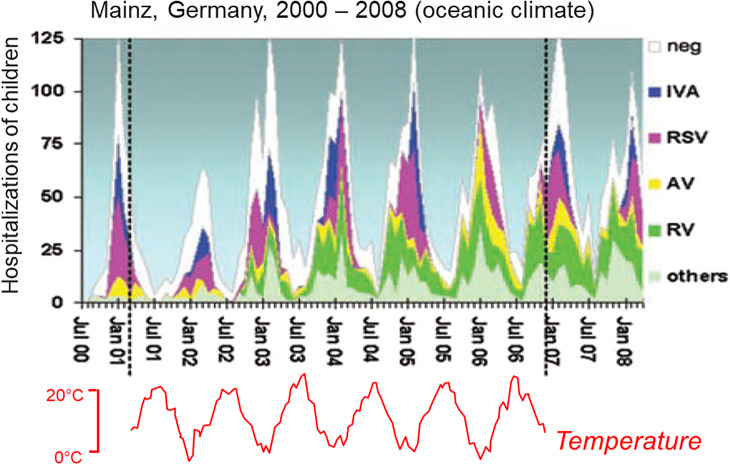
Case numbers of children aged 16 years or below hospitalized by various DRVs in Mainz, Germany, 2000–2008. The four most frequent pathogens were, in order, rhinovirus (RV), respiratory syncytial virus (RSV), influenza virus A (IVA), and adenovirus (AV). Note that rhinovirus, usually considered mild, hospitalized substantial numbers of children. The timing of all illnesses is variable. For example, RSV cases mainly occurred before 1 January in the winter of 2002/2003, but after 1 January from 2005 onwards. In spite of this variation, the peak for all hospitalizations combined was normally around February, and the minimum was around August. Adapted from du Prel JB *et al. Clin Infect Dis* 2009;49(6):861–83.

While observations show that virtually all DRVs are mild in most people, it is not obvious why this should be the case. It is interesting to speculate on the selective trends involved. It might be expected that common cold viruses would often be internalized and cause dangerous or unpleasant complications *after* the contagious period. This is similar to the argument of Markov *et al.* above when they point out that death from COVID-19 generally occurs after the contagious period, so they argue there is little natural selection to reduce host mortality. Ewald points out that we should focus on mobility rather than mortality, but the same difficulty remains: why should selection that preserves host mobility (during the contagious period) prevent complications later on? The answer seems to be that viral selection is influenced by two important factors: (1) preschool children are especially susceptible to cold viruses, having five to seven colds each year. About 10% to 15% of children will have more than 11 infections. Adults have two to three colds per year, but the incidence is increased by contact with children [15]. In a study where adults were challenged by administering nasal drops containing four common cold viruses, parents were less likely to develop cold symptoms than nonparents, suggesting that parents have had exposure to a wider range of respiratory viruses and therefore have greater immunity [16]. This suggests that children can provide a breeding ground for novel virus variants that subsequently spread to adults. (2) Almost all DRVs are more prevalent during the winter, probably because they are thermally sensitive (as discussed below). For example, Du Prel *et al.* found that the number of hospitalizations of children for respiratory illnesses in Mainz, Germany, was about six times greater in midwinter than midsummer, caused by a variety of DRVs including RSV, rhinovirus, influenza, and adenovirus (Figure 1) [17]. Since children are normally kept at home if they develop serious symptoms such a fevers, the need for respiratory viruses to spread and survive the winter in children might provide a filter that removes the more pathogenic strains that would otherwise reach the bloodstream and cause systemic infections in adults. Note that even relatively mild cold viruses such as rhinovirus frequently hospitalize young children [17] (Figure 1). Selection in young children in winter may thus promote the emergence of viruses that rarely cause serious illness in healthy older children and adults throughout the year.

## Biological factors that may support the evolution of reduced pathogenicity of direct respiratory viruses

Table 2 shows some important biological factors that may support the evolution of reduced pathogenicity.

**Table 2.** Factors that may decrease the pathogenicity of respiratory viruses

Underlying biophysical phenomena	Comment	References
Transmission of respiratory viruses takes place mainly via aerosols and droplets	Viruses can only remain airborne for limited periods, so infected hosts must normally be physically close to susceptible hosts for transmission to occur by this route. This normally requires the infected host to be mobile.	[4, 5]
Low durability on solid surfaces is correlated with mildness in respiratory viruses.	Most respiratory viruses are occasionally transmitted via solid surfaces. SARS–CoV–2 survives on solid surfaces for only a few hours. Therefore, it is dependent on host mobility for efficient transmission.	[4, 7]

Outcomes	Comment	References
Long incubation periods can increase the chance of transmission. Any arbitrary mutation that slows virus replication or increases virus thermal sensitivity could extend both incubation and contagiousness.	More pathogenic strains often have shorter incubation periods. For example, the incubation period of influenza in England in 1918 was as brief as 41 h. Antarctic studies show that the incubation periods of mild respiratory viruses can extend for several months.	[18–20]
The nose and throat are among the coldest parts of the body, while the lungs are warm. Thermal sensitivity, where a virus is more active below normal body temperature, can prevent severe illness, increase incubation periods, and preserve host mobility.	This is the temperature–dependent viral tropism hypothesis. The thermal sensitivity of SARS–CoV–2 and many other respiratory viruses can easily be demonstrated in the lab.	[21, 22]
Altered cleavage sites in the surface proteins of respiratory viruses may change the location of the virus when protein cleavage takes place. Depending on the sequence, cleavage can take place within the cell during egress, outside the cell, or within the cell during viral entry.	The spike protein of SARS–CoV–2 is cleaved within the cell by furin, which is thought to make it more pathogenic. Alteration to this site and a second site with many basic residues could alter or reduce pathogenicity.	[23]
Other biochemical changes can potentially reduce pathogenicity and preserve host mobility.	These include changes to the binding of the virus to cells, changes to cell entry, changes to transcription, and novel RNA–protein interactions.	[24–27]
Higher immunity levels in the human population may favour lineages with long incubation periods causing mild symptoms.	If only a few hosts are susceptible, low levels of parasite replication, resulting in increased mobility and shedding for longer periods, can increase the chance that an infectious host will meet and infect a susceptible host.	[5]

### Incubation periods

A pathogen may incur several costs from extremely rapid replication, including reduced host mobility and a shortened contagious period, due to a more rapid immune response or possibly host death [18]. Incubation periods of respiratory viruses can vary from less than two days to several months. Focusing first on short periods, Ewald discussed the selective pressures acting on influenza among soldiers in the trenches during World War I [18]. He noted that conditions could hardly have been more favourable for the evolution of increased virulence of airborne pathogens. Soldiers were grouped so close that even immobilized sick soldiers could transmit the infection. Moreover, additional new susceptibles would be transported to the trenches to replace the sick individuals who had been removed. Sick individuals were typically moved to crowded rooms that contained new susceptibles – incapacitated soldiers and other workers. Doctors and nurses could act as vectors, spreading the infection in a manner analogous to insects that spread arboviruses (arboviruses are generally more pathogenic than respiratory viruses [5]). Reports show that the incubation period of 1918 influenza was unusually short. In 1918, two doctors travelled from London to York, sharing a railway compartment with an airman who had severe symptoms of influenza [19]. Both doctors developed flu symptoms within 41 h, and their families caught the disease from them in a similar period. Ewald also pointed out that chicken-rearing operations offer great potential for continual transmission from severely ill individuals, much like the conditions in 1918 at the Western Front. Severe epidemics have, in fact, recurred repeatedly in chicken-rearing facilities, caused by a variety of influenza types, including H5N1, H5N2, H7N3, H7N4, and H7N7. The variety of influenza types involved suggests that the potential for lethal epidemics is generally present among influenza variants when environmental circumstances allow transmission from very sick hosts [28].

At the other extreme, some mild DRVs can be present in human hosts without causing symptoms or eliciting a strong immune response for several months. Several studies within the community have identified individuals without symptoms who, despite not having undergone seroconversion, were found to be carriers of influenza A [29–31] or B [32]. Thai *et al.* commented that this ‘may indicate that viral RNA remained in the respiratory tract without being internalized and eliciting an immune response’. Several studies have detected cold viruses for extended periods without host symptoms, including a study of rhinovirus [33]. Muchmore *et al.* reported persistent parainfluenza virus shedding at the South Pole by healthy young adults throughout the 8½-month winter isolation period. Two episodes of respiratory illness caused by parainfluenza occurred after 10 and 29 weeks [20]. A two-year study of respiratory infections caused by eight common respiratory viruses in New York City found that over 70% of the infections detected by a multiplex PCR assay were asymptomatic [34]. Influenza and human metapneumovirus caused more symptomatic infections (50% and 70%, respectively). Any mutation that slows up viral replication, including mutations that reduce binding to cells, affect RNA secondary structure, reduce RNA interactions with the nucleocapsid protein, reduce RNA replication or transcription, or increase thermal sensitivity, as well as mutations to cleavage sites, has the potential to increase incubation periods and reduce pathogenicity.

### Thermal sensitivity

Like many or most other respiratory viruses, SARS-CoV-2 has been shown to be thermally sensitive in the wet lab and animals, meaning that it is more active below normal body temperature [22]. Scientific awareness of the importance of viral thermal sensitivity dates back to at least 1959, when the Nobel laureate André Lwoff [35] noted that the degree of virulence of viruses is related to their temperature sensitivity, with greater sensitivity to heat being correlated with reduced virulence. This sensitivity of SARS-CoV-2 is apparent in the laboratory: in separate studies, the attachment of SARS-CoV-2 to cells, its entry into cells, viral transcription, and liquid-liquid phase separation of nucleocapsid protein (bound to specific genomic RNA elements) were shown to be greater below normal body temperature [24–27]. Recent studies show that the Omicron subvariants bind human ACE2 more strongly at lower temperatures, and that the affinity for ACE2 spikes from newly emerged subvariants including BA.2.86 is associated, particularly at low temperatures, with their growth rates [36, 37]. Similar thermal sensitivity has been observed for influenza A H1N1, H3N1, H3N2, H7N1, influenza C, rhinovirus, parainfluenza virus, and human metapneumovirus [21, 38]. Since the nose and throat are normally colder than the lungs, such thermal sensitivity can prevent the replication of viruses in the lower airways, reducing the severity of respiratory infections. (Thermal sensitivity may also explain the seasonality of virtually all respiratory viruses since the temperatures in the nose and throat fall when air temperatures fall during the cold season [21].) Note that measles and similar viruses (as well as the viruses that cause hand foot and mouth disease) also seem to possess thermal sensitivity because they have winter seasonality and they replicate in the skin, which is usually below normal body temperature, causing rashes. It is also interesting that insect-borne viruses can benefit from thermal sensitivity because they are normally transmitted via the skin. It is likely that the thermal sensitivity of respiratory viruses varies as they adapt to their local climate and season: strains that are insufficiently thermally sensitive in polar regions are likely to immobilize their hosts before they can spread, while strains that are *too* thermally sensitive in the Tropics will be unable to replicate even in the slightly colder tissues of the nose and throat [21]. Adaptation to its local climate can explain why influenza tends to move from hotter places to colder, e.g. from South China to North China [21]. Weimken *et al.* [39] found COVID-19 seasonal spikes from approximately November through April for all outcomes and in all countries investigated, which can be attributed to its thermal sensitivity. The presence of thermal sensitivity in so many respiratory viruses, including SARS-CoV-2, is most easily interpreted by assuming that selective trends tend to moderate the pathogenicity of respiratory viruses and that most respiratory viruses use temperature sensing as a convenient way to avoid immobilizing their hosts while replicating in sites in the nose and throat that allow efficient transmission [21].

### Proteolytic cleavage of surface proteins

Another important consideration is the potential for alterations to the cleavage sites of the fusion proteins on the surfaces of viruses. Many viruses, including HIV, Ebola, influenza, and coronaviruses, require such cleavage. Cleavage allows the surface proteins to enter high-energy metastable states, allowing their conformation to change rapidly, thereby fusing the viral and cell membranes. Alterations to cleavage sites can easily impact viral pathogenicity. The hemagglutinins of many influenza A viruses, including human and low pathogenic avian influenza A viruses, possess monobasic cleavage sites (R/K↓) and are typically cleaved by the transmembrane protease TMPRSS2 [23]. In contrast, the hemagglutinins of some highly pathogenic avian influenza A viruses are cleaved at a multibasic furin cleavage site (such as R-X-R/K-R↓). The lack of a furin site in both human and low pathogenic avian influenza virus is thought to moderate pathogenicity, again suggesting that natural selection may moderate pathogenicity in conditions that do not favour rapid transmission. In this respect, SARS-CoV-2 may occupy an intermediate position because the spike protein of SARS-CoV-2 is cleaved twice, first by furin or similar cellular proteases at the S1/S2 site during egress, then at a second site called the S2’ site, typically by TMPRSS2 at the cell surface. Roughly half of the known coronaviruses have furin cleavage sites at the S1/S2 junction within the spike protein, which may allow for near-complete cleavage during egress and, thus, efficient receptor binding when the virus encounters new host cells [23, 40]. A laboratory-generated SARS-CoV-2 mutant that lacked the furin cleavage site replicated at a reduced rate in a human respiratory cell line and was attenuated in both hamster and transgenic mouse models of SARS-CoV-2 [41]. Sequence variations at the S2’ site also influence pathogenicity and transmissiblity. The SARS-CoV-2 Omicron variants BA.1 and BA.2 seem to favour cell entry via endosomes and cleavage of the S2’ site by cathepsins (rather than TMPRSS2), which is believed to reduce replication in human lung cells compared to the Delta variant. However, Omicron lineage BA.5 shows increased TMPRSS2 usage compared to BA.1 and BA.2 [23]. Further changes in the cleavage sites of the spike protein clearly have the potential to influence the pathogenicity of SARS-CoV-2 positively or negatively.

## Factors that may increase pathogenicity

Table 3 lists some of the biological factors that might increase the pathogenicity of SARS-CoV-2 and other respiratory viruses. As discussed above, SARS-CoV-2 that replicates in the warmer parts of the body (lungs, lymphatics, etc.) does not normally pass back to the airways and escape as droplets and aerosols. However, replication in many parts of the body might increase viral shedding, and circumstances that favour transmission by this (systemic) route could, in principle, arise in hospitals, refugee camps, or other settings that offer the potential for continual transmission from very sick individuals. Similarly, circumstances encouraging transmission via exudates on solid surfaces could favour more pathogenic strains that increase total virus shedding, even if the shedding period is brief. Additionally, Ewald pointed out that faecal transmission, including through contaminated drinking water, could increase virulence because large numbers of susceptible people could become infected by the pathogens released by a few immobilized hosts [18]. Another danger is that the recombination of SARS-CoV-2 with animal coronaviruses might result in a strain with increased pathogenicity.

**Table 3.** Biological and behavioural factors that could increase the pathogenicity of respiratory viruses

Factor	Comment	References
‘Direct’ respiratory viruses (DRVs) such as SARS–CoV–2 could acquire lymphatic tropism.	Replication in the lymphatics can increase the incubation period of viruses. It also provides access to the bloodstream and may allow the virus to invade other organs. This may contribute to the higher pathogenicity of measles and smallpox (in smallpox, mortality was correlated with the level of viremia during the first two days).	[7, 10]
Increased transmission on solid surfaces could increase selection for durability.	A sit–and–wait strategy might become more favourable, meaning that high levels of host mobility would be less advantageous.	[7]
Increased faecal–oral transmission.	Large numbers of susceptible people can be infected by the pathogens released by a single immobilized individual via contaminated drinking water. Viruses that replicate in the gut, such as polio virus, may reach the bloodstream (and all organs).	[18]
Recombination with other animal or human viruses.	A phylogenetic analysis indicated that the virus that caused the 2009 swine–origin H1N1 influenza A epidemic was produced by the reassortment of several viruses previously circulating in swine, including H1N1 classical swine, H3N2 seasonal human, and H1N1 avian influenza viruses. Recombination with animal coronaviruses might similarly increase the pathogenicity of SARS–CoV–2.	[42]

We should also consider human activities and events that could increase the pathogenicity of SARS-CoV-2, some of which are listed in Table 4. It might be expected that the introduction of mass testing for viruses for the first time during the COVID-19 pandemic would have had an important impact on the evolution of pathogenicity. PCR testing followed by tracing of the contacts of infected individuals may have been harmful in this respect. Since there was a delay in getting an appointment for a test, and it took one or more days to receive the results, we might expect a strong selection of strains that could replicate and spread so quickly that testing and tracing could not stop them. Such aggressive strains might outrun the track and trace system, while milder strains, which might otherwise have had an advantage, might have been stopped. The effects of the instant lateral flow tests, which arrived later, are more difficult to predict. In previous pandemics, in the absence of testing, individuals infected by strains that were mild and caused ‘colds’ would have continued to socialize and spread the virus for several days. Free lateral flow tests would have prevented this in many cases. On the other hand, strains that sometimes gave completely asymptomatic infections might have been favoured, since lateral flow testing would not normally have detected them.

**Table 4.** Human activities that could increase the pathogenicity of SARS-CoV-2

Activity	Comment	References
High–volume testing.	Testing with PCR, where results are not available for one or more days, may strongly favour fast (therefore, often, pathogenic) strains. Lateral flow tests, which give rapid results, may prevent the spread of both mild and pathogenic strains, reducing the natural advantage of mild strains.	
Accidental release of modified viruses from laboratories.	The effects on pathogenicity of modifying viruses in the laboratory cannot be predicted with confidence. Strains with altered tropism might be created.	[43, 44]
Transmission by attendants and in settings where host mobility is limited, e.g., refugee camps, prisons, migrant worker dormitories, hospitals, nursing homes, barracks, and boarding schools.	Since transmission can continue in such settings even when hosts are immobilized by severe sickness, highly pathogenic strains may evolve.	[18]
Poor sanitation in developing countries	More pathogenic strains may evolve since waterborne transmission can continue even when hosts are immobilized by severe sickness. Although such specialist strains are unlikely to outcompete strains that are well–adapted to the respiratory route, recombination between strains might create variants of concern.	[18]
Social, political, or cultural pressures on human hosts to continue socializing when sick. This includes political crises such as wars.	Social pressure to remain mobile may have contributed to the extreme pathogenicity of the 1918 influenza strain.	[18]

Accidental release of pathogens from laboratories is not uncommon, and laboratory alterations to the virus, such as alterations to cleavage sites and receptor binding domains, might result in increased pathogenicity [44]. Repeated serial passage through cells is used to make viral strains for live attenuated vaccines. It tends to reduce the fitness of viruses to be transmitted naturally, reducing the danger of accidental release [45]. On the other hand, selection during laboratory passaging can be very powerful because scientists often transfer orders of magnitude more viruses to cells than are transmitted during natural infections. Therefore, adaptation in cell cultures can be very rapid, and one or a few passages in cells might give rise to strains with novel, possibly more harmful, features.

Poor sanitation in developing countries might allow a more pathogenic strain specializing in faecal transmission to evolve. However, extensive adaptation to this route would probably prevent significant transmission by the airborne route.

Any social, political, or cultural pressures for individuals to continue working and socializing when sick might increase the virulence of respiratory viruses, including SARS-CoV-2. A crisis such as a war might have this result.

Note that some changes can create ‘tipping points’ where changes to the tropism of SARS-CoV-2 occur that are unlikely to be reversed. For example, strains released from laboratories or arising from poor sanitation might stably pass through the lymphatics or evolve increased durability. By contrast, other factors, such as increased testing in a few countries, or transmission in crowded environments, might increase pathogenicity temporarily and reversibly.

## A note on vaccines

The effect of vaccination on the evolution of virus pathogenicity is unclear and may depend on the vaccine’s effectiveness. In the cultural climate that currently exists in many countries, it seems unlikely that high vaccine uptake levels will be achieved soon. Nevertheless, by reducing the number of susceptible individuals in a population, even vaccines with low uptake might support the emergence of mild strains with longer incubation periods that can better preserve host mobility. On the other hand, vaccines that are ineffective or confer antibody-based but not T-cell receptor-based immunity may promote the emergence of novel variants (possessing altered surface proteins) to which the population may be susceptible. Aggressive strains with shorter incubation periods may emerge if susceptible hosts become more abundant due to novel surface proteins.

## Conclusions

Hundreds of thousands of people are made sick by animal viruses every year. Some of these ‘spillovers’ cause haemorrhagic fevers such as Ebola, Marburg virus disease, and the Crimean-Congo and South American haemorrhagic fevers. Lassa haemorrhagic fever kills around 5,000 people each year [46]. If you go back far enough in time, virtually all present-day human respiratory viruses must be descended from viruses that spilled over to our forebears from other animals, and presumably, many were highly pathogenic at first, like some present-day spillovers. These observations suggest that selective pressures exerted during extended human-to-human transmission tend to reduce pathogenicity. Moreover, the distant progenies of some of these spillovers, those that developed ‘confined’ upper respiratory tract tropism (i.e., today’s common cold viruses), are almost universally mild, as we have seen. Four decades ago, Ewald suggested that the unusual mildness of respiratory viruses can be explained by selective pressures for their hosts to remain mobile [5]. Moreover, SARS-CoV-2 does not currently pass through the lymphatics and is not spread by the faecal-oral route. Presumably, we can, therefore, expect COVID-19 to become similarly mild, provided that the virus’s current tropism is maintained. However, these considerations do not provide a time frame – they only suggest a direction of travel. Public health records from 1839 to 2000 for England and Wales show the impact on deaths from influenza (and influenza-like illness) of five major pandemics, occurring during the years 1847–1848, 1890–1891, 1918–1919, 1957–1959, and 1968–1970 [47]. (It has been suggested that the 1890–1891 pandemic was caused by the progenitor of the human coronavirus OC43 rather than an influenza virus [48].) In most cases, the main wave lasted for one cold season (occasionally two), but multiple recurrent smaller outbreaks followed the main waves, and influenza mortality remained elevated for two to three decades after the main waves [47]. The 1918 influenza epidemic was unusual in affecting people in the prime of life, whereas a study of cases in Copenhagen showed that the waves that returned in the next two decades mainly affected individuals aged over 65, so the emergence of new strains that can attack different segments of the population can explain some of these recurrent outbreaks (suggesting that, for example, future SARS-CoV-2 variants might target children) [49].

In summary, major changes in SARS-CoV-2’s tropism are not expected, but they cannot be ruled out, so we cannot rule out the evolution of greater pathogenicity. Unfavourable scenarios include SARS-CoV-2 evolving towards passage through the lymphatic system and the bloodstream, like measles and smallpox viruses. A second danger is that the virus might replicate in the gut and be spread by a faecal-oral route, similar to the polio virus. Both scenarios require reduced thermal sensitivity, and both allow or may allow the virus to reach the bloodstream, from where it might replicate ‘by accident’ (i.e. not as a result of previous selection) in other tissues, increasing the risk of serious or life-threatening complications. Without major changes to the virus’s tropism, we should expect milder variants to emerge as SARS-CoV-2’s pathogenicity and host immunity approach equilibrium. We should, therefore, try to avoid the eventualities listed in Table 4. While a downward trajectory of pathogenicity is expected if there is no change in SARS-CoV-2’s tropism, the time scale is uncertain. Observations of previous influenza and influenza-like pandemics suggest that it may take two or more decades to become a common cold virus. We can hope that more effective medical interventions, including vaccines, will become available in a shorter timeframe.

## Data Availability

This study reviews published scientific literature, and it did not generate or use novel data.
